# Left Atrial Strain Identifies Increased Atrial Ectopy in Patients with Beta-Thalassemia Major

**DOI:** 10.3390/diagnostics11010001

**Published:** 2020-12-22

**Authors:** Maria Vlachou, Vasileios Kamperidis, Efthymia Vlachaki, Georgios Tziatzios, Despoina Pantelidou, Afroditi Boutou, Chrysa Apostolou, Despoina Papadopoulou, George Giannakoulas, Haralambos Karvounis

**Affiliations:** 1 1st Cardiology Department, AHEPA Hospital, Aristotle University of Thessaloniki, Stilponos Kyriakidi 1, 54621 Thessaloniki, Greece; mavlahoo@yahoo.gr (M.V.); geotziatzios@gmail.com (G.T.); g.giannakoulas@gmail.com (G.G.); hkarvounis@gmail.com (H.K.); 2 Thalassaemia Unit, Ippokratio University Hospital, 54642 Thessaloniki, Greece; efivlachaki@yahoo.gr (E.V.); chrisapostol@hotmail.com (C.A.); 3 Thalassaemia Unit, AHEPA University Hospital, 54621 Thessaloniki, Greece; dpantelidou@yahoo.gr (D.P.); depipap@otenet.gr (D.P.); 4 Pulmonary Department, Papanikolaou Hospital, 57010 Thessaloniki, Greece; afboutou@yahoo.com

**Keywords:** beta-thalassemia major, arrhythmia, premature atrial contractions, left atrial strain

## Abstract

Patients with beta-thalassemia major (β-ΤΜ) may develop cardiac arrhythmias through a multifactorial mechanism. The current study evaluated the association of cardiac structure and function on echocardiography with atrial ectopic burden on 24-hour tape recording in β-ΤΜ patients. This prospective study included consecutive β-ΤΜ patients. Demographic, laboratory, echocardiographic, cardiac magnetic resonance (CMR) T2* and 24-hour tape recording data were prospectively collected. The patients were classified according to the median value of premature atrial contractions (PACs) on 24-hour tape. In total, 50 β-TM patients (37.6 ± 9.1 years old, 50% male) were divided in 2 groups; PACs ≤ 24/day and > 24/day. Patients with PACs > 24/day were treated with blood transfusion for a longer period of time (39.0 ± 8.6 vs. 32.0 ± 8.9 years, *p* < 0.007), compared to their counterparts. Older age (OR: 1.121, 95% CI: 1.032–1.217, *p* = 0.007), longer duration of blood transfusion (OR:1.101, 95% CI:1.019–1.188, *p* = 0.014), larger LV end-diastolic diameter (OR: 4.522, 95% CI:1.009–20.280, *p* = 0.049), higher values of LA peak systolic strain (OR: 0.869, 95% CI: 0.783–0.964, *p* = 0.008), higher MV E/E′ average (OR: 1.407, 95% CI: 1.028–1.926, *p* = 0.033) and higher right ventricular systolic pressure (OR: 1.147, 95% CI: 1.039–1.266, *p* = 0.006) were univariably associated with PACs > 24/day. LA peak systolic strain remained significantly associated with PACs > 24/day after adjusting for the duration of blood transfusions or for CMR T2*. The multivariable model including blood transfusion duration and LA peak systolic strain was the most closely associated with PACs > 24/day. Receiver operating characteristic curve analysis identified a left atrial peak systolic strain of 31.5%, as the best cut-off value (83% sensitivity, 68% specificity) for prediction of PACs > 24/day. In β-TM patients, LA peak systolic strain was associated with the atrial arrhythmia burden independently to the duration of blood transfusions and CMR T2*.

## 1. Introduction

Beta-thalassemia major (β-TM) is a genetic disease characterized by a significant decrease or even absence of the β-globin chain, a component of normal hemoglobin A [1]. Regular blood transfusions are the cornerstone treatment in the management of β-TM, but they cause body iron overload when iron chelation treatment is not optimal [1]. Cardiac iron overload seems to be the most important factor, attributing to β-TM-induced cardiomyopathy [2].

Cardiac iron overload is the primary cause for arrhythmias in β-TM patients [1,3]. Nevertheless, increased cardiac output and dilated atria, due to chronic anemia, are the main substrates for atrial arrhythmic events in non-cardiac iron overload β-TM patients [1,3].

Premature atrial contractions (PACs) are usually observed at least once, in 24 h, in individuals>50years old and are not considered clinically significant [4,5]. Nevertheless, recent studies suggest that patients with frequent PACs are at high risk of developing atrial fibrillation [5,6].

Multi-modality imaging has a diagnostic and prognostic role in patients with cardiac arrhythmias [7,8,9,10]. A comprehensive echocardiographic examination remains the cornerstone imaging modality to evaluate a patient with cardiac arrhythmias, by detecting or ruling out structural heart disease, evaluating cardiac function, and offering implications for overall prognosis [7,8,9,10]. Nowadays, speckle tracking echocardiography has a significant role in detecting subclinical left ventricular (LV) systolic dysfunction, in assessing atrial function and has even a prognostic role, in patients with atrial arrhythmias [7,9]. Left atrial (LA) longitudinal peak systolic strain has recently been recognised as a non-invasive parameter that can assess the global atrial function, contraction and relaxation, in patients with atrial arrhythmias [7,8,9,10].

In β-TM patients there are scarce data on the echocardiographic parameters which predict atrial arrhythmias. Thus, the current analysis aimed to identify echocardiographic parameters which are associated with PACs burden in asymptomatic β-TM patients.

## 2. Patients and Methods

### 2.1. Study Population

This prospective study included all consecutive β-ΤΜ patients, systematically transfused and chelated, who were referred from the Haematology Departments to the Cardiology Outpatient Thalassaemia Clinic for their routine annual cardiology follow-up. Exclusion criteria were systolic heart failure, known coronary artery disease, atrial fibrillation, significant mitral or aortic valvular disease, congenital heart disease, severe renal or liver dysfunction, thyroid uncontrolled disorders, electrolyte imbalance, malignancies and acute or chronic inflammation diseases. The University Ethics committee approved the study protocol (Aristotle University of Thessaloniki, Medical School, Bioethics Committee, Project Identification Code: 9270, Date: 12 July 2017). All the patients, who participated in the study, provided written informed consent.

### 2.2. Study Protocol

Demographic, clinical, electrocardiographic, echocardiographic, 24-h Holter monitoring parameters, blood samples for hemoglobin, urea, creatinine, ferritin, bilirubin (total and indirect), lactate dehydrogenase and cardiac magnetic resonance (CMR) T2* assessment were prospectively recorded. All the tests were performed within 3 days before the blood transfusion.

#### 2.2.1. Conventional Echocardiography

Comprehensive 2-dimensional and Doppler transthoracic echocardiography was performed with a commercially available ultrasound system (Vivid-7 and S7, General Electric, Horten, Norway) equipped with 3.5 MHz or M5S transducers. Data were stored digitally and analyzed offline on a dedicated workstation (EchoPac 112.0.1, GE Medical Systems, Horten, Norway). All echocardiographic exams were performed by an experienced cardiologist, echocardiography specialist.

LV dimensions were measured on the parasternal long-axis view and LV mass was estimated according to the formula by Devereux et al. (0.8 × {1.04 [(LVEDD +PWTd+ SWTd)^3^− (LVEDD)^3^]} +0.6) g; where LVEDD is left ventricular end-diastolic diameter, PWTd is posterior wall thickness in diastole, SWTd is septal wall thickness in diastole [11]. LV mass indexed to body surface area was then calculated [11]. Relative wall thickness [RWT = (2 × PWTd)/LVEDD] was then estimated. LA volume was acquired from two-chamber and four-chamber views, using two-dimensional echocardiography and left atrial volume index was obtained when LA volume was indexed to body surface area [11]. Left ventricular ejection-fraction was calculated with Simpson’s biplane method [11]. Right ventricular dimensions and right atrial area were measured from the modified apical 4-chamber view [12]. Right ventricular systolic function was evaluated estimating tricuspid annular plane systolic excursion on M-mode and S’(tricuspid lateral annular systolic velocity) wave on tissue Doppler imaging [12]. Right atrial pressure was estimated by measuring the inferior vena cava diameter and its respiratory alterations [12]. Right ventricular systolic pressure was estimated from the tricuspid regurgitation maximum velocity, with the simplified Bernoulli equation, and the right atrial pressure, by applying the following formula: right ventricular systolic pressure = 4 × (Tricuspid Regurgitation maximum velocity)² + right atrial pressure) [12].

Stroke volume index was calculated by multiplying the LV outflow tract cross sectional area by the velocity time integral derived from the pulsed wave Doppler recordings acquired at that point, and then indexed to body surface area [13]. Cardiac index was estimated by multiplying stroke volume by heart rate and then indexing to body surface area [13]. Mitral inflow pulsed-wave Doppler imaging and tissue Doppler imaging at the septal and lateral mitral annulus, in 4-chamber view, was performed for evaluating LV diastolic function and filling pressures according to the recommendations for assessing diastolic function [14].

#### 2.2.2. Speckle Tracking Echocardiography

**Figure 1:**
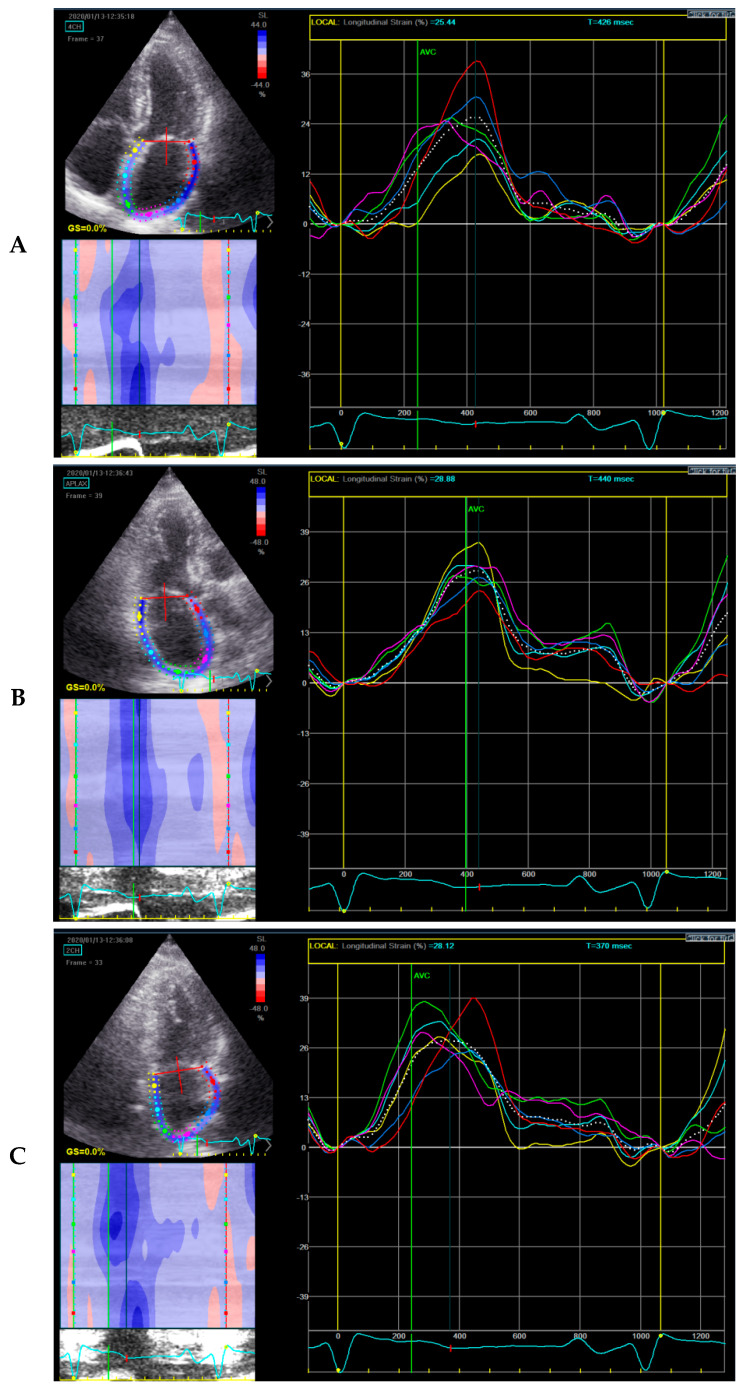
Left atrial strain by two-dimensional speckle-tracking. The area of interest in 4-, 3-, 2- chamber view can be seen in the upper left quadrant of images (**A**,**B**,**C**) respectively.

For the strain imaging based on speckle tracking echocardiography, the apical four-, two- and three-chamber views, not foreshortened and with stable frame rate, were analyzed in each patient. Aortic valve closure time was manually defined. After tracing the LV endocardial border and adjusting the region of interest to cover only the LV myocardium in apical four-, two- and three-chamber view, the system automatically calculated LV global longitudinal strain [15]. The peak positive longitudinal strain of the LA, that corresponds to the reservoir function in systole, was evaluated by speckle tracking echocardiography using the three apical views that included all LA walls. LA endocardial border was traced manually in four-, two-and three-chamber apical views and the area of interest was defined to comprise for the thin LA myocardium (Figure 1) [16,17]. The software automatically defined 6 segments of LA myocardium in each view and the peak LA strain was estimated in each apical view. Finally, the mean value of the LA peak systolic strains of the 3 apical views was determined [16,17].Of note, though Badano et al., recommended LA longitudinal strain should be calculated from apical 4-chamber view or as the mean of LA peak strain from both 4 and 2-chamber apical views, we used all the apical views for the calculation of LA strain, as per Modin et al. [16,17]. For the LA strain evaluation, the electrocardiographic referent point used was the onset of QRS wave, because using QRS as reference timing is more feasible and less time-consuming compared to the use of P wave as the reference [16,17,18].

#### 2.2.3. CMR-T2*

All patients were scanned on a 1.5Tesla MRI scanner [19]. Myocardial T2* was measured in each scan by obtaining a single short-axis mid-ventricular slice with a single breath-hold (echocardiographic-gated multi-echo technique) [19]. Myocardial T2* values <20ms were suggestive of myocardial iron overload [19].

#### 2.2.4. 24-Hour Holter Recording

All patients who were enrolled in the study underwent 24-hour Holter monitoring (Model: General Electric, Healthcare Seer 1000 Holter Recorder). Minimum, maximum and average heart rate per 24-hourswere recorded. Beats were characterized as normal, premature atrial ectopic beats and premature ventricular ectopic beats. Episodes of supraventricular tachycardias, atrial fibrillation, non-sustained ventricular tachycardias, bigeminy, couplets and triplets were recorded. 

### 2.3. Statistical Analysis

The patients were divided in 2 groups, according to the median value of PACs(24/day), which was equal to the mean, as there was a normal distribution.

Continuous variables are expressed as mean ± standard deviation, if normally distributed and as median (interquartile range) if non-normally distributed. Categorical variables were expressed as frequencies (percentage) and were compared with the chi-square (*X*^2^) test. Comparisons for continuous variables between subgroups of participants with PACs ≤ 24/day and PACs > 24/day, were performed with the independent Student’s t-test, if normally distributed, and with Mann–Whitney U test, if non-normally distributed. Univariate analysis was performed with binary logistic regression analysis and the odds ratio (OR) and 95% confidence interval (CI) were calculated and reported. The variables with a *p* < 0.1 were introduced in the multivariable model. Several multivariate models were created, each of them included only 2 parameters to avoid model overfit due to the relatively small number of events. The relative fit of each model was calculated with the −2 log likelihood and compared with the chi-square (*x^2^*).Receiver operating characteristic analysis was used to assess the value of LA peak systolic strain in predicting PACs > 24 in a 24-hour Holter recording, according to the Youden’s index (Sensitivity+Specificity−1), the sensitivity, the specificity and the area under the curve [20].

Two-tailed *p*-value < 0.05 was considered statistically significant. Statistical analysis was performed with the SPSS version 25.0 (SPSS, Chicago, IL, USA).

## 3. Results

### 3.1. Patients Characteristics

A total of 50 β-ΤΜ patients were prospectively included and divided in two groups according to the median number of premature atrial complexes; PACs ≤ 24/day (*n* = 25) and PACs > 24/day (*n* = 25).

The clinical, biochemical and 24-hour tape parameters of the 2 groups of patients are presented in Table 1. Among the clinical parameters, patients with PACs > 24/day were older (41.4 ± 8.2 vs. 33.8 ± 8.6 years, *p* = 0.002) and were transfused for a longer period of time (39 ± 8.6 vs. 32.0 ± 8.9 years, *p* = 0.007) compared to patients with PACs ≤ 24/day. In terms of biochemical parameters, the two groups were comparable. The 24-hour rape recordings revealed premature ventricular contractions but no ventricular tachycardia and no atrial fibrillation episodes. Only 4 patients were on medications that could reduce the presence of PACs (b-blocker, diltiazem, amiodarone, escitalopram), but they still had PACs > 24/day and thus they were included in the analysis.

### 3.2. Cardiac Imaging Parameters of the Patients

The cardiac imaging parameters of the 2 groups of patients are presented in Table 2. Patients with PACs>24/day had larger LV size (4.9 ± 0.4 cm vs. 4.6 ± 0.5 cm, *p* = 0.04), impaired LA function (LA peak systolic strain 30.4 ± 7.8 % vs. 37.2 ± 6.5%, *p* = 0.002), higher LV diastolic pressure based on mitral valve E/E′ ratio (8.3 ± 2.9 vs. 6.6 ± 1.8, *p* = 0.02), higher tricuspid regurgitation maximal velocity (2.4 ± 0.4 m/s vs. 2.0 ± 0.4 cm, *p* = 0.001) and higher estimated pulmonary artery systolic pressure (27.2 ± 7 mmHg vs. 21.1 ± 0.6 mmHg, *p* = 0.003) compared to their counterparts. All other echocardiographic parameters did not significantly differ between subgroups. CMR T2* values did not differ between the two groups.

### 3.3. Associates of PACs in β-TM Patients

Univariate analysis demonstrated that age (OR 1.121, 95% CI 1.032–1.217, *p* = 0.007) and duration of blood transfusion in years (OR 1.101, 95% CI 1.019–1.188, *p* = 0.014) were associated with PACs > 24/day (Table 3). The duration of blood transfusion in years was independently associated with PACs > 24/day after adjusting for other clinical or Holter parameters (Table 4).

Univariable association of echocardiography and CMR T2* parameters with PACs > 24/day in β-TM patients is shown in Table 5. Of note, LA peak systolic strain was associated with the presence of PACs > 24/day (OR 0.869, 95% CI 0.783–0.964, *p* = 0.008) (Table 5) but not with the presence of premature ventricular contractions (OR 0.95, CI 0.88–1.02, *p* = 0.13). LA peak systolic strain and right ventricular systolic pressure are the only two echocardiographic parameters independently associated with PACs>24/day after adjusting for CMR T2* (Table 6). The model including the LA peak systolic strain is more strongly associated with PACs > 24/day compared to the model including right ventricular systolic pressure (Table 6).

**Figure 2:**
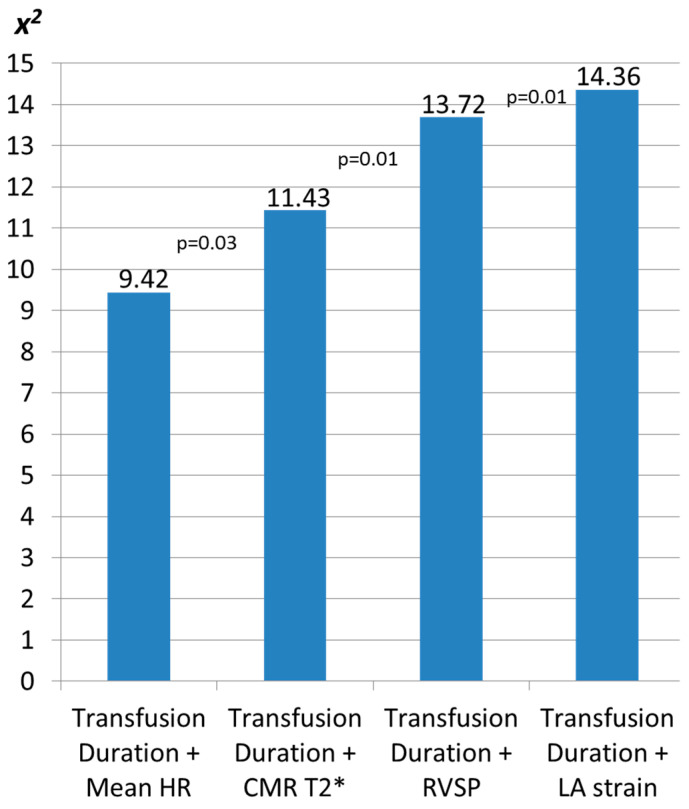
Chi-square (*x^2^*) for comparing multivariable models of clinical (blood transfusion duration, years) and Holter (mean heart rate HR, beats per minute) or cardiac magnetic resonance (CMR T2*, msec) or echocardiography parameters (right ventricular systolic pressure, RVSP, mmHg and left atrial systolic strain, LA strain, %) identifying left atrial ectopy with PACs>24 in β-TM patients. Bar graphs represent the *x^2^* of each model. The model most associated with left atrial ectopy included the duration of blood transfusions and the LA strain.

Among the multivariable models including the duration of blood transfusion in years and the mean heart rate of the 24-hour tape or the CMR T2* or the right ventricular systolic pressure or the LA peak systolic strain, the model including the duration of blood transfusion and the LA peak systolic strain was the most closely associated with PACs > 24/day (Figure 2). Of note, LA peak systolic strain was independently associated with PACs > 24/day after adjusting for the duration of blood transfusion in years (OR 0.89, CI 0.81–0.98, *p* = 0.03).

**Figure 3:**
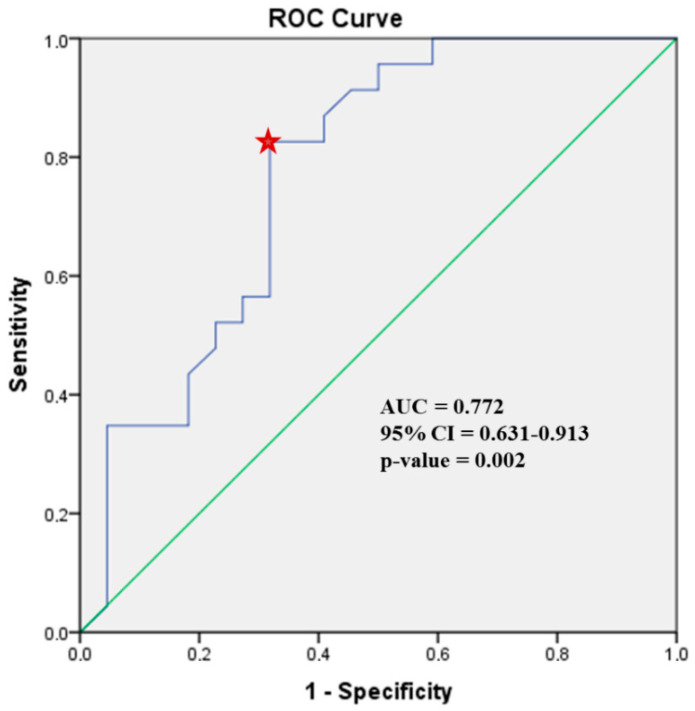
Receiving operating characteristic (ROC) curve analysis identified a left atrial peak strain of 31.5%, as the best cut-off value with 83% sensitivity, 68% specificity and Youden index 51% (red star), for prediction of PACs > 24/day (area under the curve AUC 0.772, 95% confidence interval CI 0.631–0.913, *p*-value 0.002) in β-TM patients.

Receiver operating characteristic curve analysis identified a LA peak systolic strain of 31.5%, as the best cut-off value with 83% sensitivity, 68% specificity and a Youden index of 51% (red star)**,** for prediction of PACs > 24/day (area under the curve AUC 0.772, 95% CI 0.631–0.913, *p*-value 0.002). (Figure 3).

## 4. Discussion

The present study demonstrated that in β-TM patients, LA peak systolic strain was independently associated with the frequency of PACs/day after adjustment for the duration of blood transfusions or CMR T2*. Furthermore, LA peak systolic strain ≤ 31.5% was suggested as a clinical tool to predict PACs > 24/day (sensitivity 83%, specificity 68%) in these patients.

### 4.1. Arrhythmias in β-ΤM Patients.

Cardiac arrhythmias in β-TM patients are induced through a multifactorial mechanism [1,21,22]. The main cause of arrhythmias in the above patients is iron cardiotoxicity due to iron overload as a result of the regular blood transfusions [1,21,22]. Nevertheless, β-TM patients with normal values of myocardial T2*, indicative of absence of ventricular iron overload cardiomyopathy, develop arrhythmias; atrial iron loading is thought to trigger the arrhythmias arising from the atria in the above patients [1,3]. However, in β-TM patients, the association of atrial arrhythmias with LA dysfunction, assessed by longitudinal peak strain, has never been reported before. Atrial arrhythmias are the most frequent cardiac arrhythmias in β-TM patients [1,21,23], while ventricular arrhythmias merely occur in the context of severe cardiac iron overload [1]. Accordingly, the current study demonstrated that atrial arrhythmias are associated with worse LA longitudinal strain independently of LV myocardial iron overload as expressed by CMR T2*.

Although PACs were initially considered to be benign and of low clinical significance [5], recent studies are suggestive of a correlation between PACs and the risk of development of atrial fibrillation [5,6,24]. A systematic review and meta-analysis by Himmelreich et al. showed that more frequent PACs on 24–48 h tape were related with the occurrence of atrial fibrillation and its possible deleterious effects, such as ischemic stroke, transient ischemic attack and mortality among patients without known atrial fibrillation [5,6]. Thus, in the current study, β-TM patients with more frequent PACs may be considered more prone to the development of future atrial fibrillation.

Engle et al. observed 39 β-TM patients and found that 19 patients had atrial arrhythmias (including widespread premature atrial beats, short run of supraventricular tachycardias, atrial flutter and fibrillation), 12 had ventricular premature beats and 3 patients had ventricular tachycardia [23]. In line with this, our study demonstrated that β-TM patients suffer mainly from atrial arrhythmias that were associated for the first time with impaired LA functional mechanics.

Kirk et al. showed that myocardial T2* in β-TM patients may predict the development of arrhythmias, due to myocardial iron overload [3]. Within 1 year, 83% of patients with cardiac T2* < 20 ms, and 14% of patients with cardiac T2* < 6 ms, developed arrhythmia [3]. However, the T2* values that were reported in the current study were remarkably higher than those reported by Kirk et al [3].This finding can be attributed to the fact that the current analysis included a contemporary cohort of β-TM patients who receive intensive iron chelation therapy while Kirk et al. conducted a study 11 years ago [3].Thus, the chelation therapy of the current cohort leads to normal T2* values (>30ms) and maybe this is the reason why T2* is not significantly associated with atrial arrhythmia in this study (OR 0.937, CI 0.870–1.009, *p* = 0.086).

### 4.2. LA Peak Systolic Strain and Arrhythmias

Changes in LA peak systolic strain have been observed in patients with hypertension, atrial fibrillation and LV diastolic dysfunction [25,26,27,28]. Pathan et al. determined normal mean values for LA peak reservoir strain of 39.4%, after studying 2542 healthy subjects without cardiovascular disease and comorbidities [29]. The current study of β-TM patients who had no concomitant pathologies such as arterial hypertension, chronic kidney disease, or atrial fibrillation, reported lower values of reservoir strain, reflecting the abnormal loading conditions and the LA dysfunction that are usually met in these patients.

In terms of arrhythmias, LA peak systolic strain has been studied principally in atrial fibrillation, where it was demonstrated to have a prognostic role [30,31]. Shaikh et al. found that mean LA peak systolic strain was significantly lower in patients with atrial fibrillation compared to healthy controls [30]. Moreover, improvement in LA peak systolic strain was more pronounced in patients who remained in sinus rhythm after cardioversion for atrial fibrillation compared to patients who had recurrence of atrial fibrillation [30]. Furthermore, a meta-analysis from Ma et al., including 686 patients, showed that patients with a recurrence of atrial fibrillation post catheter ablation, had lower values of LA peak systolic strain, compared to those who did not have a recurrence [31]. Last but not least, Parwani et al. showed that reduced values of LA peak systolic strain (<10%) was the most important factor associated with atrial fibrillation recurrence [32]. Hence, there is a strong association between LA peak systolic strain and atrial arrhythmia burden in the general population. To the best of our knowledge, this is the first study to correlate LA function, by evaluating LA peak systolic strain, with atrial arrhythmia burden in β-ΤM patients.

### 4.3. Study Limitations

The main limitation of this prospective study is the relatively smallcohort. However, this is a robust population of adult patients with β-TM, which is a rare fatal disease. Another limitation is that the Holter recording was only for 24 h. Additionally, no data regarding the evolution of PACs over time are provided. Thus, there is no further information of whether these patients will develop atrial fibrillation in the future. Finally, we did not repeat the echocardiography to check for any changes regarding LA strain over time. The CMR data available are restricted to the T2*.

## 5. Conclusions

In β-TM patients, LA peak systolic strain, an echocardiographic parameter indicative of LA function, is independently associated with the daily burden of PACs/day, an expression of atrial ectopic burden. All β-TM patients are followed up annually by echocardiography, thus LA strain could be used as a marker of atrial arrhythmia, resulting in rhythm monitoring with a 24-hour tape and closer follow-up of the patients with impaired LA strain. Further studies are needed to validate the role of LA strain in these patients and to elucidate its clinical significance and relation with the development of overt atrial arrhythmias.

## Figures and Tables

**Table 1:** Clinical, biochemical and 24-hour Holter parameters in β-thalassemia-major patients with premature atrial contractions ≤ 24/day and > 24/day.

	PACs ≤ 24 (*n* = 25)	PACs > 24 (*n* = 25)	*p*-Value
**Clinical Characteristics**			
Age (years)	33.8 ± 8.6	41.4 ± 8.2	0.002
Male gender, n (%)	15(60)	10 (40)	0.258
BSA (m²)	1.7 ± 0.2	1.8 ± 0.2	0.281
Blood Transfusions Duration (years)	32 ± 8.9	39 ± 8.6	0.007
Blood Transfusion Frequency (days)	15 ± 3.7	14 ± 4.6	0.16
Smoking, n(%)	8 (16)	7 (14)	0.73
Hypertension, n (%)	0 (0)	0 (0)	1
Diabetes, n (%)	3 (6)	2 (4)	1
Dyslipidemia, n (%)	1 (2)	0(0)	1
Chronic Kidney Disease, n (%)	0(0)	0(0)	1
Osteoporosis, n (%)	7(14)	7(14)	1
Splenectomy, n (%)	10 (20)	13 (26)	0.571
**Biochemical Parameters**			
Hemoglobin (g/dL)	10.2 ± 0.7	10.2 ± 0.7	0.877
Total Bilirubin (mg/dL)	2.04 ± 1.2	1.5 ± 0.7	0.059
Direct Bilirubin (mg/dL)	0.56 ± 0.23	0.42 ± 0.19	0.053
Indirect Bilirubin (mg/dL)	1.49 ± 1	1.1 ± 0.6	0.122
LDH (U/L)	258.1 ± 87	261.4 ± 116.2	0.915
Ferritin (ng/mL)	1008.3 ± 790	1037.4 ± 1198.5	0.92
**24-Hour Holter Parameters**			
Minimum HR (bpm)	59.7 ± 5.3	57 ± 6.5	0.885
Maximum HR (bpm)	126.4 ± 14.4	127 ± 18.4	0.132
Mean HR (bpm)Median value of PACs	84.1 ± 7.42 (0–9)	79.5 ± 8.8131 (61–288)	0.05<0.001
Patients with PVCs, n (%)	9 (18)	17 (34)	0.061

BSA = body surface area; HR = heart rate; LDH = lactate dehydrogenase; PAC s = premature atrial contractions; PVCs = premature ventricular contractions; SGOT = serum glutamic oxaloacetic transaminase; SGPT = serum glutamic pyruvic transaminase.

**Table 2:** Cardiac imaging parameters in β-thalassemia-major patients with premature atrial contractions ≤ 24/day and > 24/day.

	PACs ≤ 24 (*n* = 25)	PACs > 24 (*n* = 25)	*p*-Value
**Echocardiographic Parameters**			
Left Ventricular Ejection Fraction(%)	61.7 ± 4.2	62.6 ± 5.9	0.546
Stroke Volume index (ml/m²)	41.4 ± 7.6	38.4 ± 6.7	0.162
Cardiac index (L/min m²)	3.1 ± 0.6	2.9 ± 0.5	0.145
Left Ventricular End-Diastolic Diameter (cm)	4.6 ± 0.5	4.9 ± 0.4	0.04
Left Ventricular End-Systolic Diameter (cm)	3 ± 0.5	3 ± 0.5	0.712
Left Ventricular Mass index (g/ m²)	73.4 ± 16.7	82.6 ± 16.9	0.076
Relative Wall Thickness	0.36 ± 0.05	0.35 ± 0.06	0.492
Left Atrial Volume index (ml/m²)	30.5 ± 12.7	33.1 ± 10	0.422
Left Atrial Peak Systolic Strain (%)	37.2 ± 6.5	30.4 ± 7.8	0.002
Left Ventricular Global Longitudinal Strain (%)	20.5 ± 3	19.5 ± 2.4	0.206
Mitral Valve E/A velocity ratio	1.42 ± 0.4	1.41 ± 0.5	0.935
Mitral Valve E/E’ ratio average	6.6 ± 1.8	8.3 ± 2.9	0.02
Right Ventricular End-Diastolic Basal Diameter (cm)	3.2 ± 0.5	3.3 ± 0.5	0.523
Right Atrium Area (cm²)	13.1 ± 2.5	13.6 ± 2.4	0.456
Right Ventricular Fractional Area Change (%)	49.9 ± 8.7	50 ± 5.8	0.979
TAPSE (cm)	2.4 ± 0.2	2.4 ± 0.3	0.745
Right Ventricular S’ (cm/s)	15.6 ± 2.7	14.5 ± 2.3	0.103
Tricuspid Regurgitation maximal velocity(m/s)	2.0 ± 0.4	2.4 ± 0.4	0.001
Right Ventricular Systolic Pressure (mm Hg)	21.1 ± 6	27.2 ± 7	0.003
Right Atrial Pressure (mmHg)	3.7 ± 2.6	3.2 ± 1	0.389
Cardiac Magnetic ResonanceT2*			
T2* (ms)	35.6 ± 9.8	30.5 ± 9.3	0.068

A = atrial filling; E = early filling; e′ = early diastolic; PACs = premature atrial contractions; S′ = tricuspid lateral annular systolic velocity wave; TAPSE = Tricuspid Annular Plane Systolic Excursion.

**Table 3:** Clinical, biochemical and 24-hour-Holter univariate associates of premature atrial contractions > 24/day in β-thalassemia-major patients.

	Univariate Analysis		
	OR	95% CI	*p*-Value
**ClinicalParameters**			
Age (years)	1.121	1.032–1.217	0.007
Male	0.444	0.143–1.378	0.160
BSA (m²)	4.678	0.291–75.255	0.276
Blood Transfusions Duration (years)	1.101	1.019–1.188	0.014
Blood transfusion frequency (days)	0.906	0.789–1.041	0.163
Smokers	1.658	0.405–6.785	0.482
Diabetes	0.638	0.097–4.188	0.639
Osteoporosis	0.944	0.273–3.263	0.928
Splenectomy	1.773	0.571–5.507	0.322
**Biochemical Parameters**			
Hemoglobin (g/dL)	1.065	0.489–2.322	0.874
Total Bilirubin (mg/dL)	0.549	0.288–1.049	0.069
Direct Bilirubin (mg/dL)	0.064	0.003–1.257	0.07
Indirect Bilirubin (mg/dL)	0.563	0.268–1.184	0.130
LDH (U/L)	1	0.994–1.006	0.913
Ferritin (ng/mL)	1	0.999–1.001	0.918
**24-Hour Holter Parameters**			
Minimum HR (bpm)	0.932	0.850–1.022	0.134
Maximum HR (bpm)	1.003	0.969–1.038	0.882
Mean HR (bpm)	0.930	0.864–1.002	0.056
Patients with PVCs (n, %)	2.372	0.803–7.006	0.118

BSA = body surface area; CI = confidence interval; CMR = cardiovascular magnetic resonance; HR = heart rate; HR = hazard ratio; LDH = lactate dehydrogenase; OD = odds ratio; PACs = premature atrial contractions; PVCs = premature ventricular contractions; SGOT = serum glutamic oxaloacetic transaminase; SGPT = serum glutamic pyruvic transaminase.

**Table 4:** Models of clinical and biochemical or 24-hour-Holter or echocardiography or cardiac magnetic resonance T2* associates of premature atrial contractions > 24/day in β-thalassemia-major patients.

	Multivariable Analysis			Model Comparison		
	OR	95% CI	*p*-Value *	−2 log Likelihood	Chi-Square	*p*-Value ^†^
**Model 1** (Clinical)				61.709	7.61	-
Blood transfusion duration, years	1.10	1.02–1.19	0.014			
**Model 2** (Clinical + biochemical)				54.290	9.39	0.009
Blood transfusion duration, years	1.09	1.01–1.18	0.03			
Total Bilirubin, mg/dl	0.67	0.34–1.33	0.25			
**Model 3** (Clinical + Holter)				59.89	9.43	0.009
Blood transfusion duration, years	1.09	1.01–1.18	0.03			
Mean heart rate, beats per minute	0.95	0.88–1.03	0.19			

* *p*-value by multivariate logistic regression analysis; ^†^ *p*-value by likelihood ratio test vs. baseline model 1; CI = confidence interval; OR = odds ratio.

**Table 5:** Cardiac imaging univariate associates of premature atrial contractions > 24/day in β-thalassemia-major patients.

	Univariate Analysis		
	OR	95% CI	*p*-Value
**Echocardiographic Parameters**			
Left Ventricular Ejection Fraction (%)	1.036	0.926–1.158	0.538
Stroke Volume index (mL/m²)	0.941	0.864–1.025	0.165
Cardiac Output index (L/min m²)	0.427	0.134–1.364	0.151
Left Ventricular End-Diastolic Diameter (cm)	4.522	1.009–20.280	0.049
Left Ventricular End-Systolic Diameter (cm)	0.801	0.255–2.517	0.704
Left Ventricular Mass index (g/m²)	1.036	0.995–1.078	0.086
Relative Wall Thickness	0.018	0.001–1429	0.484
Left Atrial Volume index (mL/m²)	1.021	0.971–1.074	0.417
Left Atrial Peak Systolic Strain (%)	0.869	0.783–0.964	0.008
Left Ventricular Global Longitudinal Strain (%)	0.870	0702–1.079	0.205
Mitral Valve E/A velocity ratio	0.947	0.268–3.353	0.933
Mitral Valve E/E’ ratio average	1.407	1.028–1.926	0.033
Right Ventricular End-Diastolic Basal Diameter (cm)	1.535	0.423–5.565	0.514
Right Atrium Area (cm²)	1.101	0.858–1.411	0.446
Right Ventricular Fractional Area Change (%)	1.001	0.924–1.085	0.978
TAPSE (cm)	0.669	0.064–7.036	0.738
Right Ventricular S’ (cm/s)	0.820	0.644–1.045	0.108
Right Ventricular Systolic Pressure (mm Hg)	1.147	1.039–1.266	0.006
Right Atrial Pressure (mmHg)	0.857	0.590–1.245	0.418
Cardiac Magnetic Resonance T2*			
T2* (ms)	0.937	0.870–1.009	0.086

A = atrial filling; CI = confidence interval; E = early filling; e′ = early diastolic; OR = odds ratio; S′ = tricuspid lateral annular systolic velocity wave; TAPSE = Tricuspid Annular Plane Systolic Excursion.

**Table 6:** Multivariable models of cardiac magnetic resonance T2* and echocardiography associates of premature atrial contractions > 24/day in β-thalassemia-major patients.

	Multivariable Analysis			Model Comparison		
	OR	95% CI	*p*-Value *	−2 log Likelihood	Chi-Square	*p*-Value ^†^
**Model 1** (CMR)				65.59	3.72	-
CMR T2*, msecs	0.94	0.87–1.01	0.08			
**Model 2** (CMR + ECHO)				55.95	6.41	0.04
CMR T2*, msecs	0.95	0.88–1.03	0.20			
LVEDd, cm	3.89	0.85–17.85	0.08			
**Model 3** (CMR + ECHO)				60.96	8.35	0.02
CMR T2*, msecs	0.95	0.88–1.02	0.17			
E/e’ ratio average	1.37	0.99–1.90	0.06			
**Model 4** (CMR + ECHO)				55.57	11.84	0.003
CMR T2*, msecs	0.93	0.85–1.02	0.12			
RVSP, mmHg	1.15	1.04–1.27	0.008			
**Model 5** (CMR + ECHO)				50.52	12.34	0.002
CMR T2*, msecs	0.94	0.86–1.03	0.19			
LA Strain, %	0.87	0.79–0.97	0.01			

* *p*-value by multivariate logistic regression analysis; ^†^ *p*-value by likelihood ratio test vs. baseline model 1; CI = confidence interval; CMR = cardiac magnetic resonance; E = early filling; e’ = early diastolic; ECHO = echocardiography; LA = left atrial; LVEDd = Left Ventricular End-Diastolic Diameter; msecs = milliseconds; OR = odds ratio; RVSP = right ventricular systolic pressure.

## Abbreviations

β-TM	beta-thalassemia major
CI	confidence interval
CMR	cardiac magnetic resonance
E	trans-mitral early filling wave
E′	early diastolic wave
LA	left atrium/left atrial
LV	left ventricle/left ventricular
OR	odds ratio
PACs	premature atrial contractions
